# Analyzing Trends of Loneliness Through Large-Scale Analysis of Social Media Postings: Observational Study

**DOI:** 10.2196/17188

**Published:** 2020-04-20

**Authors:** Keren Mazuz, Elad Yom-Tov

**Affiliations:** 1 Hadassah Academic College Jerusalem Jerusalem Israel; 2 Microsoft Research Herzeliya Israel; 3 Faculty of Industrial Engineering and Management Technion Haifa Israel

**Keywords:** loneliness, text postings, behavior online, social media, computer-based analysis, online self-disclosure

## Abstract

**Background:**

Loneliness has become a public health problem described as an epidemic, and it has been argued that digital behavior such as social media posting affects loneliness.

**Objective:**

The aim of this study is to expand knowledge of the determinants of loneliness by investigating online postings in a social media forum devoted to loneliness. Specifically, this study aims to analyze the temporal trends in loneliness and their associations with topics of interest, especially with those related to mental health determinants.

**Methods:**

We collected a total of 19,668 postings from 11,054 users in the loneliness forum on Reddit. We asked seven crowdsourced workers to imagine themselves as writing 1 of 236 randomly chosen posts and to answer the short-form UCLA Loneliness Scale. After showing that these postings could provide an assessment of loneliness, we built a predictive model for loneliness scores based on the posts’ text and applied it to all collected postings. We then analyzed trends in loneliness postings over time and their correlations with other topics of interest related to mental health determinants.

**Results:**

We found that crowdsourced workers can estimate loneliness (interclass correlation=0.19) and that predictive models are correlated with reported loneliness scores (Pearson *r*=0.38). Our results show that increases in loneliness are strongly associated with postings to a suicidality-related forum (hazard ratio 1.19) and to forums associated with other detrimental behaviors such as depression and illicit drug use. Clustering demonstrates that people who are lonely come from diverse demographics and from a variety of interests.

**Conclusions:**

The results demonstrate that it is possible for unrelated individuals to assess people’s social media postings for loneliness. Moreover, our findings show the multidimensional nature of online loneliness and its correlated behaviors. Our study shows the advantages of studying a hard-to-reach population through social media and suggests new directions for future studies.

## Introduction

Loneliness has become a public health problem described as an epidemic in modern society [1]. Recent research has produced evidence of the widespread nature of the condition and of the long-term impact of loneliness on health [2]. Loneliness poses risks to emotional and social health and to physical well-being. More than half (55%) of lonely people were more likely to be of poor health [3]. The prevalence of suicide ideation has been found to increase with the degree of loneliness [4,5].

Loneliness is defined as a subjective and emotional experience of unpleasant response to a lack of satisfactory companionship [6]. It appears across all age groups. More than a quarter of UK adults report sometimes being lonely, and 6% of them report being lonely all or most of the time [7]. In the 2002 Health and Retirement Survey, 19.3% of US adults older than 65 years reported feeling lonely for much of the previous week [8]. A 2018 study of adults [9] found that those 18-22 years of age had the highest average loneliness score, 48.3%, of all the generations surveyed, followed by those 23-27 years of age with an average score of 45.3%. The lowest loneliness scores were among the oldest adults, those ≥72 years of age, with an average score of 38.6%.

Loneliness has been increasing in modern society [10], and there are concerns that using new online technologies and social media is contributing to rising loneliness. Previous investigations of the relationships between loneliness and media use suggested that lonely people do attempt to compensate by watching television, listening to radio, reading magazines, or going to movies [11]. However, it remains unclear whether social media provides the same relief from loneliness as these more traditional media.

Some studies suggest that loneliness can predict internet addiction [12] and problematic use of the internet [13], assuming that loneliness is an important determinant of social media use [14,15]. Dittmann [16] noted that loneliness appeared higher among participants who not only spent a lot of time online (more than 40 hours per week), but also preferred online interactions instead of face-to-face interactions or telephone communications. The heaviest internet users were more likely to demonstrate shyness, loneliness, and dissociation [17].

Loneliness has been found to be a positive predictor of Facebook addiction, standard Facebook use, and Facebook entertainment [18]. Passive social media use has also been found to be positively related to user’s depressive symptoms [19]; social anxiety [20]; increased loneliness, envy, and shame [21]; and lower life satisfaction [22]. Lou et al [23] found that there was no significant difference in loneliness between those who create social media content and those who consume it—both creation and consumption were significantly related to loneliness. Błachnio et al [24] also found that people who had a compulsion to use their phone during contact with other people more often used Facebook in an excessive way and felt lonelier and scored lower on self-esteem and satisfaction with life.

Some researchers have cautioned against internet use since it creates loneliness, but others have identified its beneficial effects on social capital [25], well-being [26], and loneliness [27].

When confronted with excessive life stress, users may use social media as a means of stress relief or as a stress-coping strategy to escape from reality and to compensate for unsatisfactory social interactions [28]. A study of first-year students revealed that social media users seek out friends to dismiss the stress that is associated with poor adjustment [29]. Thus, social media could be used to satisfy users’ need for psychological escape when confronted with real-life problems or challenging situations, especially to reduce emotional stress.

Pittman [30] found that individuals who used social media more were less likely to report being lonely. Gardner, Pickett, and Knowles [31] identified “social snacking behaviors” (such as looking at photos or rereading old emails) as symbolic social behaviors that can alleviate loneliness by serving as a reminder of existing social bonds. Clark et al [32] found that, although adolescents might be more socially (physically) isolated today, they also see less of a need for physical relationships than in the past. Previous generations grew up satisfying the need for social connection through physical relationships, but adolescents today appear to be more comfortable satisfying that need through digitally mediated activities [30].

As reviewed above, a large body of studies has been devoted to the increasing use of the internet, social media, and smartphones, and their effects on loneliness levels. Because of the diverse and even contradictory research results, such as those described above, there is a need to keep exploring the effects of digital behavior on loneliness, as these seem to have quite diverse relationships with measures of psychological well-being. Activity on social networks has been studied as a window to people’s behavior in the physical world, especially related to health and medicine [33]. For example, De Choudhury et al [34] studied transitions from discussion of depression to those of suicidal ideation on Reddit, a popular social network. Furthermore, postings on social networks have been correlated with depression [35,36]. Other work investigated tracking people’s experiences of adverse drug reactions using Twitter data [37]. Furthermore, Yom-Tov et al [38] demonstrated how physical and mental characteristics of people with anorexia could be tracked in online social networks through their postings and self-reported characteristics (both physical and mental). More generally, it has been suggested that digital traces can enrich and enhance psychological research [39] by providing data from people in their natural environment.

Discussions in online networks and online communities were also studied as a window to medical decision making. As such, several works [39,40] investigated an online forum for considerations of parents related to vaccinations. Similarly, Huber et al [41] examined discussion threads regarding treatment choices in a group for patients with prostate cancer.

The aim of this study is to expand the knowledge on the determinants and trends of loneliness via online postings in a social media forum. We provide an analysis of social media postings written by users who self-identify as lonely. Posting in a social media forum is understood here as an attempt to initiate social interaction online, and it is an integral and ongoing part of social media behavior. Thus, we first investigated whether loneliness can be assessed by an unrelated person that reads a post. Then, we studied the temporal trends of loneliness levels and their correlations with loneliness-related interest topics to understand the trajectory of posting behavior. We showed that loneliness, as expressed in social media postings, can be assessed by unrelated individuals. We developed an automated algorithm that can assess loneliness given the text of a posting and validated this algorithm both by comparing it to human-generated scores and by showing consistency in the scores of the same individuals. This enabled us to use the model to score a large number of postings on loneliness and to propose the following hypotheses.

- Hypothesis 1: Temporal trends in loneliness scores are associated with future topics of interest, including serious mental health outcomes and suicide.
- Hypothesis 2: A decrease in loneliness scores is associated with lower probability of suicide postings, compared to increasing or even constant scores.

## Methods

### Participants and Procedure

This study was an internet-based field experiment. To test our hypotheses, we chose data from the social network Reddit (reddit.com). Reddit is a social news aggregation, web content rating, and discussion website. Posts are organized by subject into user-created boards called “subreddits”, which cover a variety of topics including news, science, movies, video games, music, books, fitness, image-sharing, and loneliness. As of March 2019, Reddit had 542 million monthly visitors (234 million unique users) and was ranked as the sixth most visited website in the United States and 21st in the world, according to Alexa Internet, with 53.9% of its user base coming from the United States, followed by the United Kingdom at 8.2%, and Canada at 6.3% [42].

Reddit has all the main features of discussion forums in that users post their own personal messages and photos. Like other major blog sites, anyone can visit other users’ forums to read and post messages with simple clicks. Thus, everybody can post their personal messages knowing that others can easily access those messages. Each user is identified by a username, which can be (and often is) anonymous.

We extracted all postings made to the Reddit discussion board on loneliness (r/lonely) between January 1, 2015, and December 31, 2018 (4 years). We further extracted all posts on other subreddits made during these dates by people who posted on the loneliness discussion board. Posts made by people who subsequently deleted their account were removed from analysis.

On July 2014, a user posted a link to the long-form University of California, Los Angeles (UCLA) loneliness questionnaire and asked users to report their scores. A total of 40 users reported their scores, and, of those, 23 posted messages to r/lonely or to r/ForeverAlone (a similar subreddit). The long-form UCLA questionnaire is closely correlated with the UCLA Loneliness Scale (ULS-6) questionnaire used in our work (see Measures) [43]. We extracted these postings and reported the validity of our models by comparing self-reported scores to estimated scores, as described in the Measures sections.

### Measures

We asked 7 crowdsourcing workers on Mechanical Turk to label a random sample of 236 posts (made by 230 users) from the loneliness discussion board. Workers were shown a post, asked to imagine how they would feel had they wrote this post, and then answer the short-form ULS-6 to measure respondents’ loneliness [44]. The ULS-6 is a widely used tool [45] for gauging the loneliness of respondents. It includes six statements that people are asked to rate their agreement with. The total score is the aggregate of the agreement scores.

We then averaged the scores given by the workers for each question and calculated the loneliness score for each user. High scores indicate a higher degree of loneliness. Score reliability was estimated using interclass correlation and Krippendorff’s alpha [46].

Using the 236 labeled posts, we built a predictive model for loneliness scores given the text of a question. All words, word pairs, and word triplets that appeared in 5 or more postings and fewer than 50% of postings were used as attributes for predicting loneliness scores. Additionally, we extracted sentiment attributes from the NLTK [47] package. This package provides an estimate of the positive, negative, neutral, and composite valence of sentiment.

The two feature families (phrases and sentiment) were modeled using a random forest with 50 trees. We noted that other models, including regression trees and linear models, achieved worst performance.

The random forest model was then applied to all postings from the loneliness forum, and further analysis (ie, stratifying posts according to predicted loneliness scores) was conducted.

All analysis was conducted using Matlab 2019a. This work was approved by the Institutional Review Board of the Technion – Israel Institute of Technology.

## Results

A total of 19,668 postings from 11,054 users were found on the loneliness forum on Reddit. A further 406,714 postings were found to have been made by these users on other Reddit forums. Of the latter, 20.71% (84,249/406,714) were made after the first post in the loneliness forum and 79.23% (322,246/406,714) before it.

We first analyzed the reliability of unrelated individuals in estimating loneliness. Interclass correlation among crowdsourcing workers across the short-form ULS-6 was, on average, 0.19 (SD 0.03). Krippendorff’s alpha was, on average, 0.19 (SD 0.03). This agreement is sufficient to allow a rough estimate of loneliness to be assessed by these workers. This lends support to our assumption.

The predictive model was trained separately for each of the ULS-6 statements using leave-one-out error estimation. The Pearson correlation between the total predicted score and the score estimated using crowdsourcing was 0.36 (*P*<.001). The use of phrases (word, word pairs, and triplets) alone reached a Pearson correlation of 0.21 (*P*=.001), and the use of sentiment attributes reached 0.34 (*P*<.001).

To estimate the validity of our models, we compared self-reported loneliness scores that were available for 23 users with the scores estimated by applying the models to their postings, as described in the Methods section. The score for users who posted more than once was taken as the maximum score across posts. The Spearman correlation between the estimated and reported loneliness scores was 0.48 (*P*=.02). Thus, our model represents moderate validity.

All collected posts in the loneliness forum were then scored using the model. Approximately 20.35% (2250/11,054) of users made more than one post in the loneliness forum. The correlation between the loneliness scores of the same user at different times is Spearman ρ=0.22 (*P*<.001).

Since both annotator agreement and classification accuracy were not high, we stratified users into five percentile bands, from people whose posts received an average predicted score in the low 20% of the scores, up to the top 20%.

We analyzed the trends in loneliness postings over time for 356 users who had five or more postings in the loneliness forum. First, their predicted loneliness scores were interpolated using linear interpolation from the first time they posted and for a period of 316 days, which represents the 50th percentile of the time span between the first and last posting among these users. These interpolated scores were normalized by removing the value of the first predicted score for each user and then clustered using k-means into 5 clusters, where each user was represented by their predicted loneliness score on each day.

**Figure 1:**
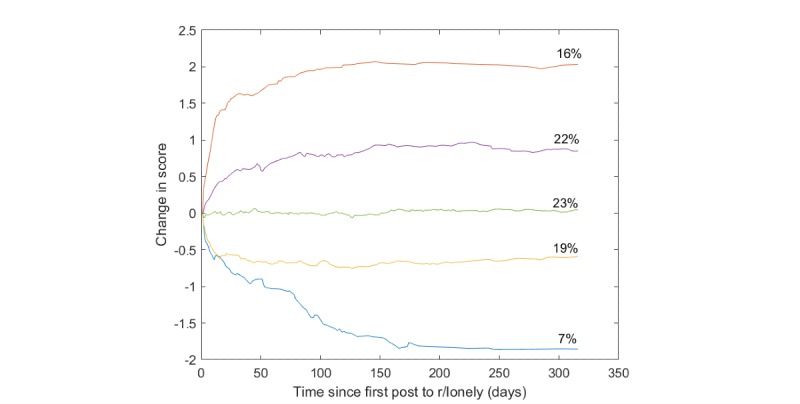
Centroids of users clustered over time by predicted loneliness scores and the percentages of users in each cluster who made posts in the suicide watch forum.

Figure 1 shows the centroids of the 5 clusters as well as the percentage of users in each cluster who asked a question in the suicide watch forum (Multimedia Appendix 1 shows the figure with error bars). The average time between a posting to the loneliness forum and to the suicide watch forum was 215 days (SD 251). The partition is nonrandom (chi-square goodness of fit test, *P*<.001). As the figure shows, a decrease in loneliness scores is associated with a lower likelihood of suicide postings, compared to increasing or even constant scores. This lends support to our second hypothesis.

Notably, users who had a large increase in their loneliness were somewhat less likely to post on the suicide watch forum. However, in contrast to other clusters, all the users in this cluster posted in the suicide watch forum prior to their first post in the loneliness forum.

We noted that people with constant scores (the central cluster) also had high transition rates to r/SuicideWatch. We hypothesize that users with constant loneliness scores still suffer from emotional vulnerability that can affect their transition to suicidal ideation, see also [34].

We further noted that, in general, loneliness scores stabilized after around 100 days. We hypothesize that, after an initial period (reflected by beginning to post on r/lonely), people’s loneliness scores stabilize and do not change significantly during the observation window.

To model the relationship between postings to the loneliness forum and future posting to the suicide watch forum, we examined users who made 2 or more posts to the loneliness forum and modeled future postings to the suicide watch forum using a Cox proportional-hazard model. The independent variables to this model were the current predicted loneliness score and the change in loneliness score. We included only cases where the time between postings on the loneliness forum was within 180 days.

Table 1 shows the model parameters for this analysis. We note that the time of day and day of the week were not statistically significantly associated with posting to the suicide forum. As the table shows, a lower current score is strongly associated with future postings to the suicide watch forum, as is a recent increase in loneliness scores. This provides support to our first hypothesis.

We modeled postings to the 50 most popular Reddit forums (excluding r/SuicideWatch analyzed above) among people who posted to the loneliness forum using the same methodology as above. Table 2 shows the model parameters for those forums where one or both variables were statistically significant at *P*<.05 using the Bonferroni correction.

**Table 1:** Cox proportional-hazard model for future postings on the suicide watch forum. N=6751.

Attribute	Exp (B)	SE	*P* value
Current score	0.775	0.102	.01
Change in score	1.195	0.079	.02

**Table 2:** Cox proportional-hazard model for future postings on different forums among the 50 most popular forums among people who posted in the loneliness foruma.

Forum	Exp (B)					Topics
	Current score	*P* value	Change in scores	*P* value		
r/depression	1.36	<.001	0.84	<.001	Depression	
r/Drugs	1.44	<.001	0.80	.008	Illicit drugs	

^a^Only forums where at least one variable was statistically significant (*P*<.05 with Bonferroni correction) are shown. The description column of topics is taken from the official description of the subreddit, or from the authors’ understanding of this subreddit, if no official description exists.

We selected users who posted to the loneliness forum and to at least 10 or more other forums. We then analyzed posts made by these users to forums that had postings by at least 50 of these users and not more than 50% of them. Users were represented by the number of posts to each forum and clustered using nonnegative matrix factorization with 10 clusters.

Table 3 shows the most important forums in each cluster, according to the strength of the latent factor. Labels for the clusters are generalizations proposed by the authors.

**Figure 2:**
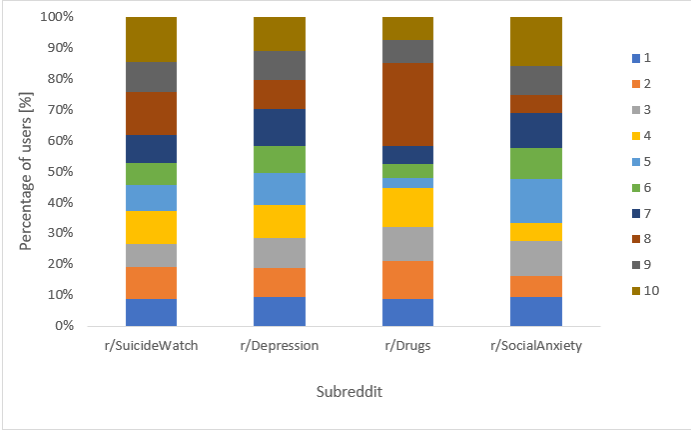
Percentage of people in each of the clusters shown in Table 2 who posted in specific subreddits. The partitions are statistically significant (chi-square test) only for r/Drugs (*P*=.049).

Interestingly, the likelihood of users in each of the clusters to post in subreddits related to the future behaviors shown in Table 2 varies significantly, as shown in Figure 2.

As the figure shows, people in cluster 8 were most likely to post about drugs. Together with people in cluster 10, they were also the most likely to post in the suicide watch subreddit. People in cluster 7 were the most likely to post in the depression subreddit. People in cluster 5 were low in all these behaviors. Thus, we deduce that people who post in the loneliness forum have diverse interests and backgrounds, and these behaviors are further associated to significantly varying degrees with interest in serious mental conditions.

**Table 3:** Most important forums in each cluster, according to the strength of the latent factors identified by nonnegative matrix factorization. Cluster titles are suggested by the authors.

Cluster (Title)	Subreddits
Cluster 1 (Depression)	r/depression r/depressed r/misanthropy r/disability r/selfharm
Cluster 2 (Leisure)	r/funny r/weddingplanning r/pics r/Atlanta r/gifs
Cluster 3 (Other)	r/3amjokes r/NoStupidQuestions r/satanism r/findareddit r/Lightbulb
Cluster 4 (Gaming)	r/leagueoflegends r/summonerschool r/TeamRedditTeams r/LeagueConnect r/formula1
Cluster 5 (Music)	r/shittyideas r/PipeTobacco r/Showerthoughts r/Blink182 r/ToolBand
Cluster 6 (Young males)	r/ForeverAlone r/virgin r/NEET r/AnxietyDepression r/Fuckthealtright
Cluster 7 (Multimedia)	r/GifSound r/Supernatural r/HFY r/dvdcollection r/FlashTV
Cluster 8 (Teens)	r/teenagers r/SonicTheHedgehog r/nukedmemes r/fakealbumcovers r/nin
Cluster 9 (Gaming)	r/Warthunder r/hoi4 r/pumparum r/shittydarksouls r/darksouls3
Cluster 10 (Illicit drugs)	r/opiates r/Stims r/OpiatesRecovery r/benzodiazepines r/trees

## Discussion

The aim of this study is to expand the knowledge on the determinants of loneliness via online postings in a social media forum. To the best of our knowledge, this is the first study to examine such relations on a large scale and with this methodology approach.

We found that there is a correlation between the total predicted loneliness score and the score estimated by crowdsourcing workers, as we assumed. The posted text can be read and assessed for its loneliness level to an acceptable degree by others, as if they were in the post writer’s shoes. This is in lieu of a self-reported assessment by the post writers themselves, which is impossible to obtain in an anonymous social network. This is particularly important considering the subjective, dynamic, and ubiquitous features and bias of loneliness online and offline that could impact how people assess their own levels of loneliness.

Having demonstrated that loneliness-related postings can be assessed, we further demonstrated that an automated algorithm could assess loneliness given the text of a posting. We validated this algorithm both by comparing it to human-generated scores and by showing consistency in the scores of the same individuals. Additionally, for a small sample of users who provided an assessment of their scores in unrelated postings, good correlation was attained with predicted scores, demonstrating validity of the text-based estimator. This enabled us to use the model to score a large number of postings on loneliness and stratify users by their interests, analyzing a total of 19,668 postings from 11,054 users in the loneliness forum of Reddit.

Our results provide several interesting observations into the correlates of loneliness. First, loneliness in the context of online posting behavior is not a single-faceted phenomenon. Although our data was collected from a single subreddit, it has a trajectory that can be followed via posting behavior. Thus, people who posted on this forum could be clustered by their interests according to other subreddits where they were active, and these were correlated with specific outcomes. This clustering suggested different population characteristics, all who are lonely, and new variables to further explore the digital behavior of lonely people.

This study also confirmed that temporal trends in loneliness scores are associated with future topics of interest, including suicide. Our results show that changes in the degree of loneliness are strongly associated with suicidality (hazard ratio 1.19) and with other detrimental behaviors such as depression and illicit drug use. We confirmed that a decrease in loneliness scores is associated with a lower likelihood of suicide postings, compared to increasing or even constant scores.

We noted that the clustering seems to imply that a rise in loneliness scores is associated with a higher likelihood of posting activity on the suicide subreddit. However, whereas the Cox model measures short-term change, the clustering shows the long-term trend. Thus, we posit that long-term increase in loneliness as well as short-term reduction in loneliness are both associated with suicide ideation. We attribute the latter to the cathartic effect, whereby the ideation to suicide may cause a reduction in negative emotions such as anxiety and, perhaps, loneliness.

This study has certain limitations that could be considered in future studies. First, we verified the validity of our measure of loneliness, obtained using crowdsourcing, through internal agreement between labelers. Future work will directly assess the ability of unrelated individuals to assess loneliness by comparing their scores with those of the individual who created the posting. Second, while crowdsourcing and the statistical model enabled an assessment of loneliness scores, future work will attempt to improve this assessment, either by using new methods of language processing or by including other information on users, such as demographics.

Another limitation of our study is in the use of a black box model based on word combinations. This makes it difficult to assess the reasons that a post is deemed to be associated with high or low levels of loneliness. Such an understanding could contribute to designing interventions, for example, responses that could reduce levels of loneliness and increase windows of opportunity between the first post on loneliness and the first post on suicide. Further research in this area should include gender, age, and other cultural variables, such as internet, social media and smartphone addiction, comparison between posting pictures and texts, and comparison between text posting in forums and other online activities such as shopping and information seeking. Another natural avenue for future work is an intervention (either manual or automatic) in the forum to reduce loneliness.

Despite these limitations, we believe that this research can improve our understanding of the impact of the internet and social media on social lives and the ubiquitous emotional phenomenon of loneliness. Our model can be compared across cultures.

## Appendix

Multimedia Appendix 1 Error bars.

## Abbreviations

UCLA: University of California, Los Angeles
ULS-6: UCLA Loneliness Scale
