# Supplementary figures and images for: Analyzing Trends of Loneliness Through Large-Scale Analysis of Social Media Postings: Observational Study
Source: JMIR Ment Health. 2020 Apr 20;7(4):e17188. doi: 10.2196/17188 (PMC7199140; doi:10.2196/17188)

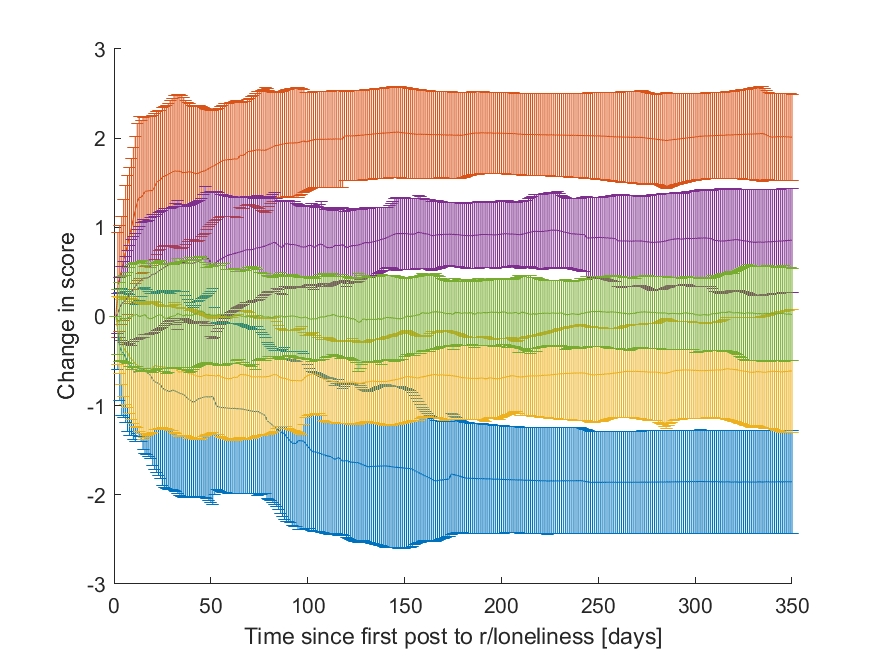


Figure S1: Error bars for Figure 1.

Supplement: Multimedia Appendix 1 [file mental_v7i4e17188_app1.docx]
